# Association between social support and viral load in adults on highly active antiretroviral therapy – Witbank, South Africa

**DOI:** 10.4102/safp.v62i1.5139

**Published:** 2020-12-03

**Authors:** Temnewo M. Habte, Charles Bondo, Lushiku Nkombua

**Affiliations:** 1 Department of Family Medicine, Faculty of Health Sciences, University of Pretoria, Pretoria, South Africa

**Keywords:** HIV, perceived social support, adherence, viral load, HAART

## Abstract

**Background:**

There are significant number of patients who are on highly active antiretroviral therapy (HAART) not virally suppressed, which is a huge clinical challenge. Social support as a non-pharmacological factor, which may influence the viral suppression, is less studied and has equivocal results. The aim of this study was to investigate the association between social support and viral load (VL) in adults on HAART.

**Methods:**

This was an analytical cross-sectional study. Using a structured questionnaire, 380 adults (≥ 18 years) on HAART for ≥ 6 months were recruited between November 2018 and February 2019 from Witbank hospital and surrounding clinics. Multivariable logistic regression was carried out.

**Results:**

The mean age of the participants was 40.5 years (s.d. = 10.3). The majority were females (73%), at least high school educated (84%), unemployed (57%), single (63%) and did not have comorbidity (80%). The vast majority had moderate to high adherence (84%) and moderate to good perceived social support (94%). The viral suppression rate was 87%. Both adherence (*p* < 0.001) and social support (*p* = 0.017) were significantly associated with VL. However, only adherence was predictive of viral suppression in multivariable analysis. Compared to poorly adherent, moderately (OR = 2.8; 95% CI = 1.32–5.98) and highly (OR = 5.3; 95% CI = 2.41–11.81) adherent participants were more likely to have suppressed VL.

**Conclusion:**

Viral suppression rate was high. Self-reported adherence to HAART was highly predictive of viral suppression, which highlights the importance of assessing and addressing adherence issues at every contact with patients taking HAART. Good social support did not predict viral suppression.

## Introduction

South Africa (SA) has the highest burden of the human immunodeficiency virus (HIV) epidemic in the world. According to the Joint United Nations Programme on HIV and AIDS (UNAIDS), 37.9 million people were living with HIV in 2018 globally.^1^ Eastern and Southern Africa is the most affected region with 20.6 million people living with HIV (PLHIV) in the same year,^1^ of which more than a third are from SA alone.^1,2^ Deaths related to acquired immunodeficiency syndrome (AIDS) were estimated to be 71 000 in SA in 2018, which is a huge decline from 140 000 deaths in 2010.^1^ This decline in AIDS-related deaths is largely attributed to the advances in highly active antiretroviral therapy (HAART).

Highly active antiretroviral therapy refers to the use of a combination of three or more antiretroviral drugs with the main goal of achieving viral suppression.^3^ In 2018, SA had about 4.7 million PLHIV on HAART, which is the largest in the world.^1^ However, there are a significant number of PLHIV who are not virally suppressed, and this is a huge challenge. In 2018, only 54% of PLHIV were virally suppressed in SA.^1^ Achieving the UNAIDS global target of 90-90-90 (90% of PLHIV know their status, 90% of those diagnosed with HIV are on HAART and 90% of those on HAART to have viral suppression^1,3,4^) in the year 2020, especially the last 90, has been an extremely difficult target. Identifying and addressing the non-pharmacological factors, which may influence the viral suppression, would be a vital adjunct to HAART.

Social support, being such a factor, is less studied and has equivocal results. In a Canadian study in the late 1990s and early 2000s, Burgoyne^5^ found that patients with better social support were more likely to have suppressed viral load (VL); however, although it was a prospective study over a 4-year period, the sample size was only 34 patients. A recent study done in Brazil showed patients with satisfactory social support had a significant reduction in VL.^6^ It was a cohort study with 1-year follow-up in which they analysed the effect of social support on quality of life, CD4 count and VL of PLHIV. They also found that in patients with unsatisfactory social support, there was no significant reduction in their VL.

Randomised control trials (RCTs) assessing different forms of support have shown mixed results. In a United States (US) study, adults with HIV VL > 1000 copies/*μ*L and/or a CD4 count < 350 cells/*μ*L were randomised to a community health worker (CHW) intervention group or control group. At 9 and 12 months, the intervention group had significantly lower VL than the control group.^7^ In a Vietnamese study, patients during initiation of HAART were randomised to a peer support intervention group or control group and were followed up for 2 years. After 2 years, peer support to improve adherence did not show any impact on the virologic and immunologic outcomes.^8^ The Vietnamese study was similar in the design and results (especially VL) to the study done in Cape Town, SA. In this SA study, patients during initiation of HAART were randomised to intervention (partial directly observed therapy [DOT] administered by community-based, patient-nominated treatment supporters) group or control (self-administered HAART) group and were followed up for 2 years. They found no significant differences in viral suppression between the two groups.^9^

A systematic review and meta-analysis of the literature, assessing the effect of directly observed therapy of HAART (DOT-HAART) on virologic, immunologic and adherence outcomes, has found that DOT-HAART has a significant effect on VL, CD4 count and adherence outcomes compared to control group; however, when analysis was done to RCTs only, DOT-HAART had no significant effect on VL.^10^ To add to the controversy, another systematic review and meta-analysis, assessing the effect of home-based interventions on virologic outcomes in adults receiving HAART in Africa, has found insufficient data to say there is a difference in viral suppression at 12 months in the home-based group compared to the standard care group.^11^

There are different forms of social support, as described by Streeter et al.,^12^ one of which is perceived social support (the individual’s perceived connections to others and his or her level of confidence that in times of need support will be available). Another one is enacted social support (received support), which is the specific actions provided by others as a gesture of support or assistance. The source of support can be formal (from professional services) or informal (from family and friends).^12^ Some of the aforementioned studies assessed perceived social support whilst others assessed what is considered as enacted social support provided by formal and informal sources of support. Different forms of social support have conflicting or different clinical outcomes on HIV VL. The evidence on the influence of social support on clinical outcome of adults on HAART is not clear cut. The primary aim of the current study is to investigate the association between perceived social support and VL amongst adults on HAART attending Witbank hospital and surrounding clinics in Emalahleni subdistrict, Mpumalanga, Republic of South Africa. Other factors (demographic, comorbidity and HAART adherence) that may influence the VL and have an effect on multivariable analysis were also explored.

## Methods

### Study design

This was an analytical cross-sectional study.

### Participant selection

The study was conducted at the wellness clinic in Witbank hospital, which is a provincial tertiary hospital, and six other surrounding primary healthcare clinics located in Emalahleni, Mpumalanga. The Wellness clinic is a specialised HIV clinic, accepting referrals of patients with multiple comorbidities and those with virologic failures. The target population was all adults who were on HAART for 6 months or more. Adults who were 18 years or older and had a VL result were included in the study. Those who did not have VL results but were due for their routine VL monitoring were also included (blood was taken for VL and results traced using a barcode). Those who did not have VL results in which results could not be traced and those who did not consent were excluded from the study. A consecutive sampling method was used. All adults who met the inclusion criteria during the study period between November 2018 and February 2019 were recruited by the researcher and a trained research assistant until the required sample size was met.

### Measurements

A structured questionnaire was used to assess the following variables: the demographic data including a question on comorbidities, self-reported medication adherence using the eight-item Morisky medication adherence scale (MMAS-8), perceived social support using the Multidimensional Scale of Perceived Social Support (MSPSS) and the current VL result.

The MMAS-8 is a valid and reliable tool to assess self-reported medication adherence.^13^ The tool contains eight questions, the first seven with yes or no reply (score of 1 for yes and 0 for no) and the last question with a five-point scale (dichotomised into score of 0 for never or rarely and 1 for once in a while, sometimes, usually, or all the time). Thus, the MMAS-8 score ranges from zero to eight. High adherence was classified as score of 0 (responding in affirmative to all indicators of adherence), moderate adherence as score of 1 or 2 and poor adherence as score of greater than 2.

The MSPSS is also a well-validated and reliable tool for assessing perceived social support.^14^ The MSPSS contains 12 questions (participants were asked to indicate how they feel about each statement), assessing the perceived support from family (e.g. : ‘My family really tries to help me’), friends (e.g.: ‘I can count on my friends when things go wrong’) and significant other (e.g.: ‘There is a special person who is around when I am in need’). Each question has seven possible replies (very strongly disagree, strongly disagree, mildly disagree, neutral, mildly agree, strongly agree and very strongly agree, from one to seven consecutively). The perceived social support was classified into a mean score of 1 to 2.9 as poor support, a score of 3 to 5 as moderate support and a score of 5.1 to 7 as good support.

The recent (less than 1 year old) VL status of each participant was also collected. Based on the national department of health guidelines and WHO recommendations, patient’s VL should be monitored at 6 and 12 months upon initiation of HAART and then annually.^3,15^ Treatment failure (virological failure) is defined by a persistently detectable VL of > 1000 copies/mL after at least 6 months of using HAART.^3,15^ For the purposes of this study, VL of ≤ 1000 copies/mL were classified as suppressed and VL of > 1000 copies/mL as unsuppressed VL.

VL testing was performed by the South African National Health Laboratory Services (NHLS) and was quantified using the automated cobas® 6800/8800 Systems (Roche Molecular Diagnostics, Pleasonton, CA, USA), which is an in vitro nucleic acid amplification test for the quantitation of HIV type 1 (HIV-1) in EDTA plasma of HIV-1-infected individuals. The test can quantitate VL (HIV-1 ribonucleic acid [RNA]) over the range of 20–10 000 000 copies/mL.

The questionnaire was directly administered and completed with help of a trained research assistant when translation was needed. Data collection and entry were double-checked thoroughly to ensure avoiding measurement errors.

### Data analysis

Initial analysis included mean, minimum, maximum and the standard deviation given for age. The frequency counts and percentages were performed to describe the demographic characteristics of the participants.

To determine an association between categorical variables of interest and VL, a Pearson’s Chi-squared test or a Fisher’s exact test was undertaken where a *p* ≤ 0.05 suggests a statistically significant association.

Multivariable logistic regression was also performed to evaluate the factors that may predict the viral suppression. Factors which were found to have significant (*p* < 0.25) association with VL were included in the multivariable logistic regression analysis. All analysis was carried out using STATA^®^ 15 (Stata Corporation, College Station, Texas, USA) and was evaluated at 5% level.

A sample size of 375 was required to achieve the objectives of the study, with 95% confidence level and 5% margin of error.

### Ethical consideration

Approval was sought from the hospital’s CEO and also University of Pretoria’s Faculty of Health Sciences ethics committee, reference number: 415/2018. A written informed consent was obtained from each participant and confidentiality was also adhered to as the information given was only used for this study purpose. The participants were identified only by a code number.

## Results

A total of 380 eligible participants from Witbank hospital and surrounding primary healthcare clinics consented and were included in the study. Table 1 illustrates the frequencies and percentages of the demographic and categorical variables.

**TABLE 1:** Frequencies and percentages of demographic and categorical variables.

Variable	Frequency (*n* = 380)	Percentage (%)
**Age**		
**Mean (s.d.)**	**40.48 (10.27)**	
18–29	51	13.42
30–39	146	38.42
40–49	117	30.79
≥ 50	66	17.37
**Gender**		
Male	103	27.11
Female	277	72.89
**Education**		
No school	17	4.47
Elementary	45	11.84
High school	269	70.79
College/university	49	12.89
**Employment**		
Employed	154	40.53
Unemployed	215	56.58
Retired	11	2.89
**Marital status**		
Married	102	26.84
Single	239	62.89
Divorced	7	1.84
Widow/widower	32	8.42
**Other medical condition**		
No	303	79.74
Yes†	77	20.26
**Adherence**		
High	187	49.21
Moderate	134	35.26
Poor	59	15.53
**Perceived social support**		
Good	179	47.11
Moderate	180	47.39
Poor	21	5.53
**Latest VL**		
Suppressed‡	329	86.58
Unsuppressed§	51	13.42

† , Includes hypertension (17.1%), diabetes mellitus (3.2%), tuberculosis (1.3%), epilepsy (1.1%), asthma (0.79%), rheumatoid arthritis (0.26%), depression (0.26%), eczema (0.26%) and deep vein thrombosis (0.26%). Of note, 4.2% had two or more other medical conditions.

‡ , VL ≤ 1000 RNA copies/mL.

§ , VL > 1000 RNA copies/mL.

VL, viral load; s.d., standard deviation.

As shown in Table 1, the mean age of the participants was 40.48 years (s.d. 10.27) and the majority were females (72.89%). In terms of educational level, 83.68% of the participants were either high school (70.79%) or college/university (12.89%) educated. Only 40.53% of them were employed; the remaining 59.47% were either unemployed (56.6%) or retired (2.9%). With regard to their marital status, most of the participants were single (62.89%), the married were 26.84% and the remaining were either widow/widower (8.42%) or divorced (1.84%).

The majority of the participants did not have additional medical condition (79.7%). With regard to self-reported adherence to HAART, 84.47% of the participants were adherent, but 35.26% were moderately adherent and 49.21% were highly adherent. The great majority of the participants (94.50%) had social support, of which 47.39% had moderate perceived social support and 47.11% had good perceived social support. Regarding VL status, 86.58% of the participants were virally suppressed (Table 1).

Table 2 illustrates the association between demographic variables and VL. The demographic factors, namely, age, gender, educational level, employment status and marital status, were all not associated with VL.

**TABLE 2:** Association between categorical variables and viral load.

Variables	VL				*p*
	Suppressed (*n* = 329)		Unsuppressed (*n* = 51)		
	*n*	%	*n*	%	
**Age**					0.303
18–29	41	12.46	10	19.61	-
30–39	126	38.30	20	39.22	-
40–49	101	30.70	16	31.37	-
≥ 50	61	18.54	5	9.8	-
**Gender**					0.157
Male	85	25.84	18	35.29	-
Female	244	74.16	33	64.71	-
**Education**					0.505
No school	15	4.56	2	3.92	-
Elementary	40	12.16	5	9.8	-
High school	235	71.43	34	66.67	-
College/university	39	11.85	10	19.61	-
**Employment**					0.770
Employed	133	40.43	21	41.18	-
Unemployed	187	56.84	28	54.9	-
Retired	9	2.74	2	3.92	-
**Marital status**					0.485
Married	92	27.96	10	19.61	-
Single	203	61.7	36	70.59	-
Divorced	7	2.13	0	0	-
Widow/widower	27	8.21	5	9.8	-
**Other medical conditions**					0.900
No	262	79.64	41	80.39	-
Yes	67	20.36	10	19.61	-
**Adherence**					< 0.001
High	173	52.58	14	27.45	-
Moderate	115	34.95	19	37.25	-
Poor	41	12.46	18	35.29	-
**Perceived social support**					0.017
Good	164	49.85	15	29.41	-
Moderate	148	44.98	32	62.75	-
Poor	17	5.17	4	7.84	-

VL, viral load.

Of the 329 virally suppressed participants, 52.58% were highly adherent to HAART, and 49.85% had good social support (Table 2). In the unsuppressed group, 37.25% had moderate adherence followed by poor adherence (35.29%), and 62.75% had moderate social support. There was a statistically significant association between adherence and VL (*p* < 0.001) and between social support and VL (*p* = 0.017) (Table 2).

Table 3 presents the factors associated with suppressed VL using multivariable logistic regression. All variables with *p* < 0.25 using the Fisher’s exact test were included in the model. Only adherence was found to be statistically significantly associated with suppressed VL. Moderately and highly adherent participants were 2.8 and 5.3 times more likely to have a suppressed VL when compared to poorly adherent participants.

**TABLE 3:** Factors associated with suppressed viral load using multivariable logistic regression model.

Factors	OR	95% CI	*p*
**Gender**			
Male	1	-	-
Female	1.9	0.98–3.76	0.057
**Adherence**			
Poor	1	-	-
Moderate	2.8	1.32–5.98	0.007
High	5.3	2.41–11.81	< 0.001
**Social support**			
Poor	1	-	-
Moderate	1.1	0.32–3.61	0.901
Good	2.6	0.73–9.35	0.141

OR, odds ratio; CI, confidence interval.

## Discussion

The relationship between different forms of social support and VL is inconclusive. Our results show that, in a bivariate analysis, perceived social support is significantly associated with VL; however, a multivariable analysis shows that perceived social support is not independently associated with viral suppression. Cunha et al. in Brazil, assessing the effect of social support on quality of life, CD4 and VL, found that those with a satisfactory social support had significant reduction in VL.^6^ Randomised control trials done by Cuong et al.^8^ in Vietnam and Nachega et al.^9^ in SA, assessing impact of peer support on virologic outcome, have both found no association between intervention and control group; whilst another RCT done by Kenya et al., assessing impact of CHW on clinical outcome (CD4 and VL), found that intervention group had significant lower VL than control.^7^

A meta-analysis and systematic review by Hart et al., assessing the effect of DOT-HAART on VL, CD4 and adherence, found DOT-HAART had a significant effect on VL, CD4 and adherence compared to the control group.^10^ However, when analysis was done to RCTs only, DOT-HAART had no significant effect on VL. In contrast, another systematic review and meta-analysis by Chishinga et al., assessing the effect of home-based interventions on VL in adults on HAART in Africa, found insufficient data to say there is a difference in viral suppression at 12 months in the home-based group compared to the standard care group.^11^

It is important to point out that the aforementioned studies used different methodology, support assessment tools and form of support. When assessing the association between perceived social support and depression in HIV patients, Sule et al. in Nigeria used similar methodology and the same social support tool and found no association between perceived social support and VL.^16^ However, their study was not primarily designed to assess this association.

The mean age of the participants was 40.5 years (s.d. = 10.3). Age was not associated with viral suppression, in keeping with findings of a study done in Ga-Rankuwa, SA, by Mogosetsi et al.^17^ However, some studies have shown that younger age of less than 35 years was associated with unsuppressed VL,^18,19^ whilst older age was more likely to achieve suppressed VL.^20,21^ In our study, females were in the majority (72.9%). This finding is similar to the local and regional literatures.^9,17,21,22,23^ The finding may be explained in two ways: the adult HIV prevalence in females is much higher than in males in SA (26.3% vs. 14.8%) as reported in the fifth SA national HIV prevalence, incidence, behaviour and communication survey;^2^ in addition, more women than men living with HIV are engaged in care in SA (60% vs. 43%) as reported by Bor et al.^4^ The association between gender and viral suppression is equivocal. This study has found no association between gender and viral suppression; this concurs with some studies.^17,24^ However, other studies have shown that male gender was associated with unsuppressed^18,22^ and suppressed VL;^20^ whilst yet in other studies, female gender was associated with unsuppressed VL.^19,21^

The study found that a huge number of the participants (83.7%) were at least high school educated. Nonetheless, educational level was not associated with viral suppression. This finding concurs with studies done in SA,^17^ Spain^25^ and Uganda.^26^ In contrast, other studies have shown lower educational level to be associated with unsuppressed VL.^19,27,28^ Most of the participants were unemployed. This finding reflected what has been reported by the authorities in SA. There was high unemployment rate of between 26.7% and 27.6% from the first quarter of 2018 to the first quarter of 2019 in SA.^29^ Employment status was also not associated with viral suppression, in agreement with the studies done in SA,^17^ US^19^ and Uganda.^26^ In terms of marital status, the majority of our participants were single and there was no association with viral suppression. Studies in SA by Mogosetsi et al.^17^ and in Uganda by Kazooba et al.^26^ have also found that marital status was not associated with viral suppression. However, in the multinational HIV Prevention Trials Network, Eshleman et al. reported that being married was associated with unsuppressed VL, although the association was weak (*p* < 0.04).^28^

The majority (80%) of our participants did not have additional medical conditions. Of those who had additional medical conditions, hypertension was the most comorbid condition. Additional medical conditions (comorbidity) were not associated with viral suppression. This finding is consistent with a recent study done in Khayelitsha, SA, by George et al., who assessed the association between non-communicable diseases (NCDs) and detectable VL and found no association between them.^21^ It is important to note that George et al. defined detectable VL as > 40 copies/mL, unlike in our study where unsuppressed VL is defined as > 1000 copies/mL.

The UNAIDS had set a global target of 90-90-90 to curb the HIV epidemic by the year 2020. By mid-2015, Mpumalanga Province (where our study population resides) had achieved 84-58-70.^30^ Our study had shown a viral suppression rate of 86.6%, which was well on track to achieve the last 90 (90% of patients on HAART to have suppressed VL) target by the year 2020. In order to achieve and maintain viral suppression, high levels of adherence to HAART is essential. A decline in the level of adherence to between 70% and 89% has been shown to be associated not only with viral rebound (unsuppressed VL) but also with resistance to HAART.^31^ Our data have shown that the higher the adherence level, the more likely the VL is suppressed. This finding is supported by multiple studies in different parts of the world.^18,19,24,32,33,34^ The finding may not be surprising. As one would expect, if the medication was not taken or taken erratically, it would not have the desired effect on the virus.

### Limitations

Besides the inherent limitations of a cross-sectional study, the study assessed participants who presented to the clinics only, patients who defaulted HAART for a longer period or who were too sick to attend the clinics may have been missed. Trained assistants (nurses) helped with translation when needed; therefore, participants may have responded to questions in a socially acceptable manner instead of responding frankly. Moreover, because of the reliance on self-reporting, response bias needs to be taken into consideration. Despite these limitations, the authors believe the findings of the study have some relevance in discussions regarding the association between social support and VL amongst adults on HAART.

### Conclusion and recommendations

The study has found that the vast majority (94.5%) of the participants had moderate (47.4%) to good (47.1%) perceived social support. The majority of the participants also had moderate (35.3%) to high (49.2%) self-reported adherence. In this study, the viral suppression rate was high (86.6%).

Although the study has found that there was significant association between perceived social support and viral suppression, good social support did not predict viral suppression. Self-reported adherence to HAART was significantly associated with VL and was highly predictive of viral suppression.

The study has highlighted the importance of adherence to HAART, as self-reported adherence was highly predictive of viral suppression. Therefore, assessing and addressing adherence issues at every contact with patients taking HAART is highly recommended in order to minimise treatment failures. Good perceived social support did not predict viral suppression in this study. However, as the perception of social support may change over time, a prospective cohort study is needed to assess the change in perception of social support and its effect on VL.
